# Association of long working hours with accidents and suicide mortality in Korea

**DOI:** 10.5271/sjweh.3890

**Published:** 2020-09-01

**Authors:** Hye-Eun Lee, Inah Kim, Hyoung-Ryoul Kim, Ichiro Kawachi

**Affiliations:** 1 Korea Institute of Labor Safety and Health, Seoul, Republic of Korea; 2 Department of Social and Behavioral Sciences, Harvard T.H. Chan School of Public Health, Boston, MA, United States; 3 Department of Occupational and Environmental Medicine, College of Medicine, Hanyang University, Seoul, Republic of Korea; 4 Department of Occupational and Environmental Medicine, College of Medicine, The Catholic University of Korea, Seoul, Republic of Korea

**Keywords:** depression, injury, karoshi, KNHANES, Korean National Health and Nutrition Examination Survey, mental health, occupational, overwork, work hour, working time, work time

## Abstract

**Objectives:**

The deleterious health effects of long working hours have been previously investigated, but there is a dearth of studies on mortality resulting from accidents or suicide. This prospective study aims to examine the association between working hours and external-cause mortality (accidents and suicide) in Korea, a country with some of the longest working hours in the world.

**Methods:**

Employed workers (N=14 484) participating in the Korean National Health and Nutrition Examination Survey (KNHANES) were matched with the Korea National Statistical Office’s death registry from 2007–2016 (person-years = 81 927.5 years, mean weighted follow-up duration = 5.7 years). Hazard ratios (HR) for accident (N=25) and suicide (N=27) mortality were estimated according to weekly working hours, with 35–44 hours per week as the reference.

**Results:**

Individuals working 45–52 hours per week had higher risk of total external cause mortality compared to those working 35–44 hours per week [HR 2.79, 95% confidence interval (CI) 1.22–6.40], adjusting for sex, age, household income, education, occupation, and depressive symptoms. Among the external causes of death, suicide risk was higher (HR 3.89, 95% CI 1.06–14.29) for working 45–52 hours per week compared to working 35–44 hours per week. Working >52 hours per week also showed increased risk for suicide (HR 3.74, 95% CI 1.03–13.64). No statistically significant associations were found for accident mortality.

**Conclusions:**

Long working hours are associated with higher suicide mortality rates in Korea.

Among the Organization for Economic Cooperation and Development (OECD) countries, Korea ranked as one of the top nations for longest working hours between 2008 and 2018 (1). In 2018, the annual working hour average in Korea was 1993 hours, while the working hour average for the OECD countries collectively was 1734 hours per year (1). Previous studies have established an association between long working hours and adverse outcomes, including coronary heart disease (2), stroke (3), mental health disorders (4, 5), reproductive health problems (6), and accidents (7). As working hours in East Asian countries (Japan, Korea, and Taiwan) are generally longer than those of western countries, deaths related to overwork (called *karoshi*), usually from cardiovascular disease, represent a growing social concern (8). Recently, suicide among overworked employees has drawn urgent attention in both Japan and Korea (9, 10). However, studies on working hours and suicide are limited to descriptive case series (11, 12), with one notable exception of a longitudinal study in the UK (13). Although the mechanism linking long working hours and suicide is not yet fully understood, a number of studies have examined the association between long working hours and depressive symptoms or suicide ideation (4, 5, 14). The deleterious impact of long working hours on mental health status is an obvious pathway connecting long working hours and suicide.

Besides cardiovascular disease and suicide, accidents are another potentially fatal outcome associated with long working hours. Fatigue and sleep loss potentially mediate the association between long working hours and accidents both in and out of the workplace (15, 16). However, the majority of studies on working hours and accidents have remained cross-sectional and/or used self-reported accidents as the outcome (7). On the contrary, a recent prospective study using national registers to assess accidents concluded there was no association between long working hours and accidents (17).

Thus, although some previous studies support an adverse impact of long working hours on suicide and accident mortality, this association is not well established by longitudinal data. In addition, to our knowledge, the association between long working hours and suicide or accident-related deaths has not been previously reported in the East Asian context.

Accordingly, the aim of this prospective study was to investigate the relationship between long working hours and accident mortality/suicide in a Korean working population based on nationwide longitudinal data.

## Methods

### Study population

**Figure 1:**
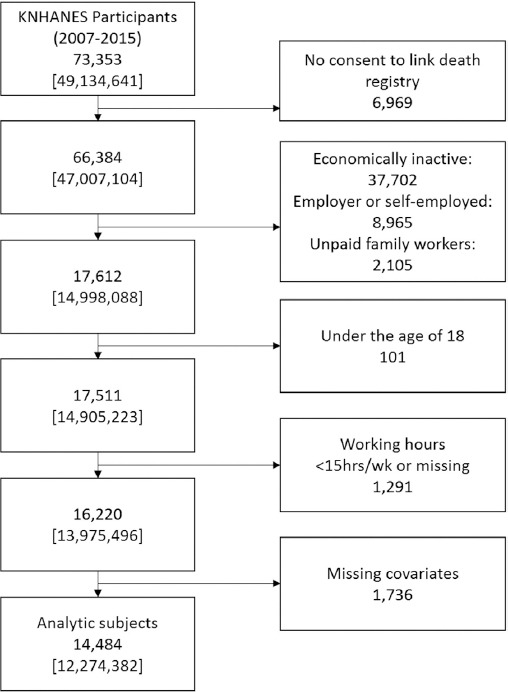
Flow chart illustrating the process of creating the cohort. Weighted frequencies showed in [ ]. [KNHANES=Korean National Health and Nutrition Examination Survey.]

Our data were derived from the Korean National Health and Nutrition Examination Survey (KNHANES) conducted by the Korea Centers for Disease Control and Prevention (KCDC) between 2007–2015. These data were then matched with death registry data compiled by the Korea National Statistical Office (KNSO) from 2007–2016. The survey used a multi-stage, cluster-sampling design based on the National Census Registry; hence, statistical analyses of this survey were based on sample weights assigned to sample participants. Among the 73 353 participants in KNHANES, 66 384 participants provided consent to link their data to the death registry. We restricted the subjects to employed workers by excluding the economically inactive population (37 702), employers and self-employed workers (8965), and unpaid family workers (2105). Employers and self-employed workers were excluded due to their ability to control their working hours; despite their working hours being even longer than employed workers, they are not subject to working hour regulations (18). Additionally, we excluded the following individuals: those <18 years, individuals with <15 work hours per week or missing information on working hours, and covariates. After these exclusions, our analytic cohort comprised 14 484 men and women. The selection process of the study population is presented in figure 1.

### Ascertainment of outcomes

The cohort dataset was matched with the death registry of the KNSO from 2007–2016 with the use of a unique identification number. As all deaths in Korea are reported to the KNSO by law, coverage of the death registry can be considered complete. Information on the specific cause of death according to the Korean Classification of Disease (KCD) and date of death was provided by KNSO. The KCD is compatible with the International Classification of Diseases-10 (ICD-10). Deaths from total external cause (V01–Y98), subsets including accidents (V01–V99; transport accidents, and W00–X59; other external causes of accidental injury), and intentional self-harm (X60–X84) were used as our outcomes. During an average 5.2 person-years of follow-up, 56 participants died from total external causes. Among them, 25 individuals died from accidents (13 from transport accidents and 12 from the other accidents) and 27 died from suicide.

### Assessment of working hours

Working hours were measured by responses to a question on the KNHANES asking: “How many hours do you usually work per week, including overtime?” Working hours were classified into four groups: (i) 15–34, (ii) 35–44, (iii) 45–52, and (iv) >52 hours per week. The top code of >52 hours per week was based on the maximum permitted working hours according to the Labor Standard Act in Korea (19). This Act has defined standard working hours as 40 hours per week, with extensions up to 52 hours per week permitted with the worker’s consent. However, working on weekends was not subject to regulation until 2018, therefore enabling workers to work >52 hours per week legally if they worked on a Saturday or Sunday.

### Covariates

Age, sex, household income, education, occupation, and depressive symptoms were included in our regression models as possible confounders. Socioeconomic status (SES), including occupation, is associated with both accident and suicide mortality (20). Depressive symptoms are a well-established risk factor for suicide and could be related to accidents as well (21). These covariates were collected during interviews in the KNHANES.

Monthly household income was equalized for household size (gross monthly household income divided by the square root of household size) and participants were divided into four groups according to quartile of standardized household income by survey year. Occupation was coded into nine categories according to the Korean Standard Classification of Occupation (22), and we collapsed these into six groups (managers and professionals; office workers; service and sales workers; agricultural, forestry, and fishery workers; plant and machine operators and assemblers; and elementary occupations). The response to the question, “Have you experienced serious sadness or hopelessness that restricts your daily life continuously for >2 weeks in the last year?” was used to define depressive symptoms, with an affirmative response indicating a positive for depressive symptoms.

### Statistical analysis

Cox proportional hazards models were developed to estimate hazard ratios (HR) with 95% confidence intervals (CI) for the association between working hours and deaths from accidents and suicide. In the Cox models, person-days were calculated from the initial date of participation in the KNHANES until either the date of death (including deaths from non-accidental causes) or 31 December 2016, whichever occurred first. The analytic model included age, sex, education, occupation, household income, and depressive symptoms as covariates. We applied the integrated survey weights, calculated by averaging weights over sampled years, because we used data from multiple waves of the survey. The sampling weights for each wave of the survey was calculated and provided by the KCDC to ensure the survey data could be inflated to the population level from which the sample was derived. More KNHANES sampling weight details can be found elsewhere (23).

### Ethical approval

The Institutional Review Board of the Korea Center for Disease Control and Prevention reviewed and approved the pilot study of the KNHANES-linked cause of death data (IRB No. 2018-07-01-P-A).

## Results

The distribution of working hours according to sample characteristics is presented in table 1. Of these participants, 35.6% worked 35–44 hours per week, 24.7% worked 45–52 hours per week, and 24.9% worked >52 hours per week. Working for >52 hours per week was prevalent among men (30.9%), those with middle lower household income (29.1%), those with middle school education (30.5%), and plant and machine operators and assemblers (39.1%).

**Table 1:** Distribution of working hours by participants’ characteristics.

	Working hour (hours/week)											

	<35			35–44			45–52			>52		
			
	Frequency	Weighted frequency	%	Frequency	Weighted frequency	%	Frequency	Weighted frequency	%	Frequency	Weighted frequency	%
Sex												
Male	700	643 292	8.7	2560	2 407 664	32.4	2120	2 081 378	28.0	2417	2 300 438	30.9
Female	1658	1 176 870	24.3	2703	1 960 773	40.5	1280	951 084	19.6	1046	752 882	15.6
Age (years)												
<30	308	336 460	25.7	345	412 326	31.5	233	289 634	22.1	202	270 383	20.7
30–39	325	309 596	9.3	1235	1 224 546	36.7	892	955 240	28.6	742	848 280	25.4
40–49	496	380 507	11.1	1593	1 253 001	36.6	1102	916 887	26.7	1018	877 490	25.6
50–59	521	410 580	15.4	1272	1 010 361	37.9	741	606 713	22.8	762	635 506	23.9
≥60	708	383 019	24.9	818	468 204	30.5	432	263 989	17.2	739	421 662	27.4
Household income												
Lowest	441	306 118	31.9	333	259 144	27.0	203	158 863	16.6	292	234 535	24.5
Middle lower	696	560 595	18.0	1103	935 014	30.1	787	708 762	22.8	1056	906 891	29.1
Middle higher	677	541 500	13.0	1719	1 495 082	35.8	1153	1 070 957	25.6	1168	1 069 844	25.6
Highest	544	411 948	10.2	2108	1 679 197	41.7	1257	1 093 880	27.2	947	842 050	20.9
Education												
Elementary school	483	281 609	27.8	447	273 103	26.9	252	155 429	15.3	468	304 506	30.0
Middle school	292	220 770	22.0	370	282 408	28.2	245	192 832	19.3	392	305 364	30.5
High school	932	816 664	16.8	1835	1 604 593	33.0	1153	1 096 765	22.6	1407	1 339 308	27.6
≥College	651	501 119	9.3	2611	2 208 333	40.9	1750	1 587 436	29.4	1196	1 104 142	20.4
Occupation												
Managers, professionals	499	396 325	13.3	1463	1 213 594	40.8	924	810 068	27.2	599	555 315	18.7
Office workers	191	162 675	6.2	1508	1 263 301	48.2	866	772 384	29.5	466	422 802	16.1
Service & sales workers	649	543 268	24.7	779	657 864	30.0	463	419 953	19.1	651	575 142	26.2
Agricultural, forestry & fishery workers	12	6113	10.3	28	20 729	35.0	16	14 544	24.5	21	17 921	30.2
Plant & machine operators and assemblers	208	179 670	7.2	710	661 038	26.5	696	676 172	27.1	985	974 518	39.1
Elementary occupations	799	532 111	27.6	775	551 912	28.6	435	339 341	17.6	741	507 622	26.3
Depressive symptom												
No	2013	1 564 971	14.2	4761	3 972 522	36.0	3099	2 780 318	25.2	3060	2 731 800	24.7
Yes	345	255 191	20.8	502	395 916	32.3	301	252 144	20.6	403	321 520	26.3
Total	2358	1 820 162	14.8	5263	4 368 438	35.6	3400	3 032 462	24.7	3463	3 053 320	24.9

The number of cases and participant mortality rates are shown in table 2. The accident mortality rates were 16.4, 21.1, 46.7, and 36.3 per 100 000 in the <35, 35–44, 45–52, and >52 hours/week groups, respectively. Suicide rates of 12.5, 12.0, 51.2, and 52.8 per 100 000 were observed in the <35, 35–44, 45–52, and >52 hours/week groups, respectively. The majority of deaths from accidents (24 cases) were among men; there was only one case among women. Suicide rates were also higher among men (46.8 per 100 000) than women (10.2 per 100 000).

**Table 2:** Accident and suicide mortality rates by characteristics of study population.

	Person-years	Person-years (weighted)	Deaths (frequency)			Deaths (weighted frequency)			Mortality rate per 100 000 (weighted)		
		
			Total external cause	Accidents	Suicide	Total external cause	Accidents	Suicide	Total external cause	Accidents	Suicide
Total	81 927.5	66 991 485	56	25	27	44 285	20 529	21 665	66.5	30.8	32.5
Weekly working hours											
<35	12 532.6	9 269 924	6	3	2	2981	1507	1150	32.4	16.4	12.5
35–44	29 509.3	23 456 408	11	7	4	7699	4910	2790	33.0	21.1	12.0
45–52	19 389.3	16 801 464	19	7	11	17 491	7818	8567	104.6	46.7	51.2
>52	20 496.3	17 463 689	20	8	10	16 114	6294	9158	93.0	36.3	52.8
Sex											
Male	44 284.6	40 854 353	48	24	22	40 001	19 752	19 004	98.5	48.7	46.8
Female	37 642.8	26 137 132	8	1	5	4284	777	2660	16.5	3.0	10.2
Age (years)											
<30	4919.8	5 690 569	1	1	0	489	489	0	8.8	8.8	0.0
30-39	18 028.1	18 331 998	11	4	7	10 921	3780	7141	59.9	20.7	39.2
40-49	24 553.7	19 206 355	18	9	9	17 636	8854	8781	92.2	46.3	45.9
50-59	18 837.2	14 909 515	10	3	5	8799	3460	3709	59.2	23.3	25.0
≥60	15 588.7	8 853 048	16	8	6	6440	3946	2033	73.0	44.7	23.0
Household income											
Lowest	7511.6	5 444 165	10	1	8	6426	777	5326	118.1	14.3	97.9
Middle lower	20 820.5	17 351 384	16	9	5	11 464	7103	3698	66.8	41.4	21.5
Middle higher	26 475.1	22 518 180	15	10	5	12 485	7978	4508	55.8	35.6	20.1
Highest	27 120.2	21 677 755	15	5	9	13 910	4671	8133	64.5	21.7	37.7
Education											
Elementary school	9592.7	5 739 575	8	2	4	3195	989	1359	55.8	17.3	23.7
Middle school	7345.3	5 555 209	10	6	4	6575	4201	2375	120.2	76.8	43.4
High school	30 523.0	27 077 154	18	11	6	17 208	10 050	7020	64.0	37.4	26.1
≥College	34 466.5	28 619 547	20	6	13	17 307	5290	10 911	60.8	18.6	38.3
Occupation											
Managers, professionals	19 474.0	16 016 850	7	1	6	6365	848	5517	39.9	5.3	34.6
Office workers	16 922.9	14 027 171	9	3	5	7372	2058	4208	52.7	14.7	30.1
Service & sales workers	14 442.0	12 112 973	6	4	2	7279	4504	2775	60.7	37.6	23.2
Agricultural, forestry & fishery workers	453.0	320 893	0	0	0	0	0	0	0.0	0.0	0.0
Plant & machine operators and assemblers	14 965.3	13 869 286	14	10	4	10 627	7579	3047	77.1	55.0	22.1
Elementary occupations	15 670.4	10 644 311	20	7	10	12 643	5541	6117	119.8	52.5	57.9
Depressive symptom											
No	72 817.6	60 113 630	50	22	25	39 674	18 424	19 682	66.4	30.8	33.0
Yes	9109.9	6 877 854	6	3	2	4612	2106	1982	67.4	30.8	29.0

Table 3 shows the results from the Cox regressions examining the association between working hours and mortality due to accidents and suicide. Proportional hazards assumptions were met. In the model adjusting for sex, age, household income, education, occupation, and depressive symptoms, participants working 45–52 hours/week showed elevated total external cause mortality risk (HR 2.79, 95% CI 1.22–6.40) compared to the reference group reporting 35–44 hours/ week. Men and women working >45 hours/week showed higher suicide mortality risk (45–52 hours: HR 3.89, 95% CI 1.06–14.29; >52 hours: HR 3.74, 95% CI 1.03–13.64) compared to the reference group. No statistically significant associations were found for accident mortality.

**Table 3:** Accident and suicide mortality risk according to working hours. Cox proportional hazard model. [HR=hazard ratio; CI=confidence interval.]

	Working hours	Adjusted HR ^a^	95% CI
Total external cause			
	<35	0.94	0.29–3.04
	35–44	Reference	
	45–52	2.79	1.22–6.40
	>52	2.04	0.88–4.72
Accidents			
	<35	0.82	0.22–3.14
	35–44	Reference	
	45–52	1.78	0.57–5.52
	>52	0.98	0.32–2.98
Suicide			
	<35	0.95	0.11–8.39
	35–44	Reference	
	45–52	3.89	1.06–14.29
	>52	3.74	1.03–13.64

a Adjusted by age, sex, household income, education, occupation and depressive symptom.

## Discussion

### Total external causes

We found that individuals working 45–52 hours per week have a higher statistically significant risk of external cause mortality compared to those working 35–44 hours per week. Those working >52 hours showed a higher HR, but the result was not statistically significant. The risk of total external cause mortality is mainly driven by the excess risk of suicide because suicide showed a significantly elevated HR in the groups working 45–52 and >52 hours. On the contrary, those working >52 hours showed a lower risk of accidents compared to the standard working hour group. This opposite direction of association between accidents and suicide among the >52 hours group suggests that mortality from these causes might have different pathways.

### Accidents

In previous studies (24, 25), an adverse impact of long working hours among hospital workers (including young doctors) on traffic accidents have been reported [odds ratio 2.3 for extended shift (>24 hours), 95% CI 1.6–3.3]. In one case-crossover study (16), there was a strong trend in increased rate ratios (RR) for traffic accidents and shift duration (RR 0.92, 95% CI 0.52–1.62 for >8 hours/day, RR 4.00, 95% CI 0.45–35.8 for >12 hours/day). For work-related accidents, several studies have also revealed the association between long working hours and increased self-reported or objectively confirmed work-related injury (26, 27). One case-crossover study showed that the risk for work-related injury in workers who worked >64 hours per week was 1.88 times greater than among those who worked ≤40 hours (28). A probable explanation for the association between long working hours and accidents is fatigue due to lack of sleep (24, 29).

The current study’s results were not consistent with these previous findings. The HR for accident mortality was lower among the >52 working hour group than the standard working hour group, although the differences were not statistically significant, and the CI was wide. A number of reasons could underlie this discrepancy. First, in the current study, there was a wide time gap between the assessment of working hours and accidents, while previous works measured working hours at the time of accidents (16, 24, 25). Sleep loss and fatigue can be more related to long working hours immediately preceding the accidents. Second, the outcome of the current study was mortality from accidents, while most previous studies used experiences of accidents as an outcome. As we used an extreme end of an accident outcome, the results could not be compared directly. In fact, a previous study using a similar design to ours (census-based longitudinal study in UK) found lower or similar risk of all accidental mortality for men working >55 compared to 35–40 hours/week among professional/managers, self-employed, and routine occupations (13).

### Suicide

Although extensive research has been conducted on the association between long working hours and mental health (including depressive symptoms and suicidal ideation), very few studies have focused specifically on completed suicide. In Korea and Japan, where overwork-related suicide is a growing social concern, descriptive characteristics of suicide cases (compensated as work-related mortality) have been reported (11, 12). The daily working hours of 22 work-related suicide cases in Japan ranged from 10–16 hours (11). In a Korean report, “chronic long working hours” was the second most prevalent reason, following “acute stressful events”, for approved cases of compensable work-related mental disease, which included suicide (12). One UK-based longitudinal study examining the association of long working hours and completed suicide showed a 1.23–1.24 times higher risk in the >55 hour/week group compared to the 35–40 hour/week group among professionals/managers, but the results were not statistically significant (13).

Elevated risk of suicide might be due to the well-established association between long working hours and poor mental health (4, 5). However, suicide rate was not associated with depressive symptoms at our data baseline. This could be caused by the time gap between the survey and the events of suicide or depression. Indeed, a longitudinal study in the UK, which reported no depressive symptoms at baseline, showed a higher risk of incident depression among participants with long working hours after a 5-year follow-up (4).

A second explanation for the association between long working hours and suicide could be the deleterious effects these long hours have on relationships with family and friends. Social isolation and family conflict are widely reported risk factors for suicide (30), and long working hours have been shown to increase work–life conflict (31, 32).

According to a 2018 psychological autopsy report of the Korean Psychological Autopsy Center, among the 103 suicide cases, occupational stress was second only to mental health issues as a primary stressor. (33). In this report, qualitative analysis of 52 employed workers’ pathways to suicide revealed that their main occupational stressors included change of work, work demands, and relationships in the workplace (33). Long working hours are closely related to work demands, and work demands could affect the relationships between supervisors and coworkers.

Low SES is also a risk factor for suicide. A previous study in Korea revealed that suicide risk is 2.28 times higher in Medicaid recipients than in 10^th^-decile highest income individuals (34). In the current study, the lowest household income group showed markedly higher suicide rates (97.9 per 100 000) than other groups (20.1–37.7 per 100 000). Since working hours could be confounded by SES, we built analytic models adjusting for SES. However, we found that the association persisted in the adjusted model, suggesting that long working hours are associated with suicide risk, regardless of SES.

### Strengths and limitations

The present study has significant strengths and limitations. Its strengths are that the subjects were drawn from a nationwide sample rather than from selected subgroups. Additionally, cause of death was determined from validated records. To our knowledge, this is the first study investigating the impact of long working hours on accident and suicide mortality in Korea.

Limitations of our study must be mentioned as well: the number of cases was relatively small; therefore, the CI for HR remained wide. Especially for women, accident mortality was extremely rare. Due to the small number of the cases, caution is warranted in generalizing the results of the current study. Working hours were measured based on self-report and collected only once at baseline. Since working hours are time-dependent variables, we cannot rule out the misclassification of exposure during follow-up. This possibility of non-differential working hour misclassification could have biased the results toward the null. Other time-varying covariates such as depressive symptoms could have changed during follow-up as well. However, due to the scarcity of repeated assessments of mental health status, we were unable to conduct a mediation analysis (ie, to check whether changes in depressive symptoms mediated the association between long working hours and suicide). Nevertheless, the lack of mediation analysis does not affect overall risk estimates of working hours for outcomes. Further analysis of working hour effects on mortality from accidents and suicide with a sufficient number of cases with longer follow-up periods, larger cohorts, and additional measures of working hours and covariates may follow in the future.

### Concluding remarks

In conclusion, our study shows that workers who work long hours (>44 hours per week) have a higher risk of suicide in Korea.
